# Influence of Preoperative Foveal Layers' Thickness on Visual Function and Macular Morphology by Phacovitrectomy for Epiretinal Membrane

**DOI:** 10.1155/2022/1895498

**Published:** 2022-08-25

**Authors:** R Zhmurin, L Grajewski, L Krause

**Affiliations:** ^1^Department of Ophthalmology of the Municipal Hospital Dessau, Academic Teaching Hospital with University Departments of the Brandenburg Medical School Theodor Fontane, Brandenburg, Germany; ^2^Charité—Universitätsmedizin, Berlin, Germany

## Abstract

**Background:**

The aim of this retrospective study with short, differently dispersed follow-up is to record the relationships between the pathologies of the individual foveal layers, measured by spectral domain optical coherence tomography (SD-OCT), and to investigate their influence on pre- and postoperative best-corrected decimal far visual acuity (BCVA) by phacovitrectomy for epiretinal membrane (ERM) in comorbidity with cataract. *Patients and Methods.* 208 eyes of 173 patients with symptomatic idiopathic ERM and moderate cataract were included.

**Results:**

In all OCT morphological stages of ERM, as well as in their combination with macular lamellar hole (MLH) and vitreomacular traction (VMT), a significant difference in the thickness of the individual fovea layers was found. In addition, the entire fovea thickening led to the proportional thickening of the individual fovea layers (*p* < 0.001). The larger the central foveolar (CFT, *R*^2^ = −0.238, *p*=0.002), maximum foveal (MFT, *R*^2^ = −0.267, *p*=0.001), and ONL thickness (*R*^2^ = −0.16, *p*=0.044) were preoperatively, the worse the initial visual acuity was at all OCT stages of ERM. This was even more significant in the presence of a tractive component in the case of MLH or VMT (*p* < 0.001). In ERM without a traction component, only ONL thickening led to significant postoperative visual acuity reduction (*R*^2^ = −0.163, *p*=0.047). The foveolar retinal thickening (CFT and MFT) of the pure ERM is directly associated with distortion of the retinal layers (*R*^2^ = 0.292, *p* < 0.001 and *R*^2^ = 0.287, *p* < 0.001) as well as with separation of the ERM (*R*^2^ = 0.168, *p*=0.034 and *R*^2^ = 0.187, *p*=0.018). When ERM was combined with tractive component, CFT, ONL, and INL thickness correlated (positively) with the integrity of ellipsoid zone (*R*^2^ = 0.342, *p* < 0.05) and external limiting membrane (*R*^2^ = 0.548, *p* < 0.001).

**Conclusions:**

ONL thickening in ERM without a tractive component serves as a limited prognostic factor of postoperative visual acuity decrease. The preoperative BCVA in the groups of ERM with traction component showed significant correlation with CFT as well as with the thickness of individual foveal layers. VMT in ERM correlates with the disintegration of the ellipsoid zone.

## 1. Background

Epiretinal membrane (ERM) is becoming increasingly important today, particularly due to improved morphological examination techniques (high-resolution spectral domain optical coherence tomography) (SD-OCT) [1, 2]. Morphologically, ERM is an avascular cell proliferation of myofibroblasts on the retinal surface with the inclusion of the fovea and can be etiologically divided into two groups. In most cases, the idiopathic (primary) ERM is found without a known cause; however, the much rarer secondary form as a result of other eye diseases. ERM usually develops from the age of 60 with a prevalence of 2–20% [1]. Symptoms usually include metamorphopsia and a reduction in visual acuity [1]. These develop in particular because of tangential retinal pulling forces [3]. Metamorphopsia is morphologically associated with important biomarkers, such as the loss of the integrity between the outer and inner segments of photoreceptor layer (ellipsoid zone) in the fovea region [4] and the foveolar thickening of the inner nuclear layer (INL) [5].

Today, SD-OCT offers the possibility of a representation of the different retinal layers, which almost corresponds to a histological retinal representation. This has become indispensable in the diagnosis of ERM. OCT imaging allowed Govetto et al. to divide ERM into 4 stages (depending on the extent of the change in fovea configuration):

  Stage 1: Preserved foveolar depression
  Stage 2: Isolated elevation of the foveola
  Stage 3: Ectopy of the inner retinal layers
  Stage 4: Pronounced disorganization of the inner retinal layers (DRIL) [6]

In addition, a combination of ERM with macular lamellar hole (MLH) or vitreomacular traction (VMT) may also occur [7], with the tractive components playing an essential role in their formation [8].

The ectopic inner foveal layers (EIFL) are due to tangential forces at development of ERM, arising in stage 3 and consisting of ectopy of INL and inner plexiform layer (IPL) over foveal surface [9]. The severity of EIFL is negatively correlated with visual acuity and metamorphopsia at ERM [10, 11].

The gold standard of therapy for symptomatic ERM is a pars-plana vitrectomy with membranectomy and peeling of the inner limiting membrane (ILM peeling) [12, 13]. However, this usually leads to cataract development in phakic eyes. Subsequent cataract surgery increases the risk of posterior capsule rupture [14]. In the retrospective studies of Pollack et al. [15] and Tranos et al. [16], combined phacovitrectomy (phacoemulsification with implantation of intraocular lens, pars-plana vitrectomy (25 gauge), membranectomy, and ILM peeling) was just as effective in terms of postoperative visual outcomes as consecutive surgery starting with cataract surgery.

However, it is difficult to assess preoperatively how the visual acuity rehabilitation will be postoperatively. In publications, preoperative OCT morphological parameters, such as maximum retinal thickness and central foveal thickness, have been investigated in the context of pars-plana vitrectomy [17–19], but with controversial results in the sense of a safe predictive factor of postoperative visual function.

This retrospective study with short differently dispersed follow-up aims to determine the correlations between the OCT morphologically measurable preoperative changes of the fovea layers and OCT-morphological stages of ERM and pre- and postoperative BCVA.

## 2. Patients and Methods

The study includes 248 patient eyes, including study group and control group. The study group retrospectively included the findings of 173 patients aged 75.1 ± 6.3 years comprising 208 eyes. In total, 78 right and 130 left eyes were examined. To date, no intraocular interventions of any kind have been performed on the patients' eyes. All had a symptomatic ERM with metamorphopsia in Amsler grid test and a moderate cataract with *N* ≤ 3, *C* ≤ 2 and *P* ≤ 1 according to Lens Opacities Classification System III (LOCS III) (Table 1) at the same time. There was no difference in cataract severity between examined groups of epiretinal membrane (*p* > 0.05). All surgeries, phacovitrectomies, were performed by two surgeons from 2015 to 2019 under general anesthesia. In the preoperative examination, the biometrics were calculated using the SRK/T formula and for an axis length of less than 21 mm, using the HAIGIS formula. After consultation, emmetropia with a residual correction of −0.1–−0.4 diopters was desired by all patients. After phacoemulsification of the lens core, aspiration of the bark masses and polishing of the posterior capsule, the monofocal acrylic posterior chamber lenses with an optical diameter of 6 mm were implanted, of which 167 were single-piece and 41 were three-part. Subsequently, a pars-plana vitrectomy (25 gauge) was performed with lifting of the posterior vitreous limiting membrane, dye-assisted membranectomy, ILM peeling, and input of SF6 gas at 46 eyes or air, respectively, at 156 eyes. Either MembraneBlue (MembraneBlue Dual^®^ from DORC) at 99 eyes or Brilliant Peel (Brilliant Peel^®^ syringe from Geuder) were used as dyes in 109 eyes. The ILM peeling leads to the removal of cells on the retinal surface that cause to envelop of the epiretinal membrane. This releases the pathogenetically important contractile forces [20]. An air endotamponade was usually used. An SF6 gas tamponade was always performed after exocryo- or endolaser coagulation to avoid of retinal detachment in the intraoperatively discovered retinal foramina.

ERM was etiologically idiopathic, combinated in 34 cases with lamellar macular hole and in 15 cases with vitreomacular traction. The preoperative examination took place 3.6 ± 1.1 days before the surgery. During the hospital stay, the following complications were observed postoperatively, such as one choroidal hemorrhage, one vitreous hemorrhage, and four endophthalmitis as well as four retinal detachments, which were treated immediately. The cases with surgery requiring complications were excluded from statistical processing. The postoperative follow-up check was carried out 3.8 ± 1.2 months postoperatively. The control group included 40 healthy eyes from 32 patients aged 74.5 ± 5.1 years.

**Figure 1:**
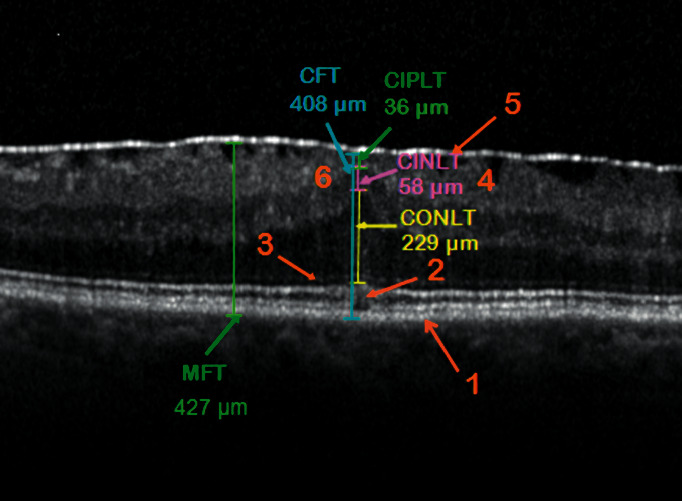
Measurement of the fovea layers in the central OCT incision in the patient's eye of Group 3. Central foveolar retinal thickness (CFT) from the retinal pigment epithelium (RPE) to the inner limiting membrane (ILM). CONLT/CINLT: central foveolar thickness of the outer and inner nuclear layer. CIPLT: central foveolar thickness of the inner plexiform layer. MFT: maximum foveal retinal thickness at the thickest fovea range from RPE to ILM. OCT morphological properties of the fovea layers: 1—intact RPE, 2—interrupted junction between the outer and inner photoreceptor segments (blurred ellipsoid zone), 3—intact integrity of the external limiting membrane, 4—distortion of the retinal layers, 5—adherent epiretinal membrane, and 6—elevation of the foveola with an ectopy of INL and IPL.

Pre- and postoperatively the BCVA was determined with the calculation of the corresponding differences. The objective refraction was determined using the Canon R-F10 Operation Manual®, the subjective refraction by means of the HAAG-STREIT Möller-Wedel visual sign system® with M3000 projector. The OCT morphological findings for the verification of ERM were recorded using the high-resolution multimodal imaging platform OCT Spectralis Spec-CAM-06961® from Heidelberg Engineering. In the central fovea area with a diameter of approximately 3 mm, morphotypic qualitative OCT properties of the retinal layers in ERM were analyzed, such as changes in the retinal pigment epithelium (RPE), integrity of ellipsoid zone, integrity of the external limiting membrane (ELM), distortion of the retinal layers, and separation of the ERM. The OCT morphological parameters were measured manually in the foveola center perpendicular to Bruch's membrane in *μ*m and labeled as follows: central foveolar retinal thickness (CFT), measured from RPE to inner limiting membrane (ILM), central foveolar thickness of the outer (CONLT), inner (CINLT) nuclear layer, and the inner plexiform layer (CIPLT). In addition, the maximum foveal retinal thickness (MFT) from RPE to ILM was measured in the central OCT macular incision. Individual measurement parameters are shown in Figure 1. All patient eyes were divided into five modified groups similar to the OCT morphological classification according to Fung et al. [6], taking into account the tractive syndromes:

  Group 1: Completely or partially preserved foveolar depression
  Group 2: Isolated elevation of the foveola
  Group 3: Elevation of the foveola with an ectopy of INL and/or IPL (EIFL)
  Group 4: MLH of tractive genesis
  Group 5: VMT

In Groups 1–3, a consistent stage-by-stage course of OCT-morphological macular changes in progression of ERM was presented, which was also described in publications by Fung et al. [6] and Mao et al. [21]. In Groups 4 and 5, a complementary tractive component is pathophysiologically mandatory. In Group 3, the thickness of EIFL was also determined.

The statistical processing for each examined group of variations was carried out within the framework of descriptive statistics using the program IBM SPSS Statistics® Version 26. An average value with standard deviation applicable to the quantitative OCT morphological parameters and a Pearson correlation coefficient (*R*^2^) were calculated. The statistical significance of the null hypothesis was determined by means of the *p*-value, each with the help of the Mann–Whitney *U* and the Kruskal–Wallis tests, depending on the number of groups.

## 3. Results

Table 2 shows the quantitative fovea parameters in absolute numbers for all morphological groups of patients' eyes compared to the control group, but with the exception of CINLT and CIPLT resulting from ectopia of the inner retinal layers. As analogues in the control group, the foveal thickness were measured via ONL to ILM at high magnification. In Groups 1–3, CFT, CONLT, and CINLT, as well as MFT, increase proportionally to the progression of ERM. These were significantly higher in VMT compared to Group 1 and the control group. As expected, CIPLT could only be measured for Group 3 with ectopy of the inner retinal layers. In ERM with MLH, CFT and CONLT are significantly lower compared to Group 1 and at the same time higher than those in the control group. Conversely, CINLT could hardly be measured in Group 4 and MFT was significantly higher compared to both Group 1 and the control group. In all groups of patient eyes with ERM, the higher values of CFT and CONLT as well as MFT could be demonstrated in relation to the control group. This speaks for an obligatory pathophysiologically causing macular thickening in ERM.

Table 3 shows the correlations between the quantitative fovea parameters mentioned above, labeling the corresponding significance level in all patient eyes with ERM. The CFT, CONLT, and CINLT, as well as the MFT, showed a positive correlation with each other, but with a diverse significance level.

The influence of the selected quantitative foveola parameters on the pre- and postoperative BCVA, as well as on the visual acuity difference, is shown in Table 4 with distortion of significance. This includes each OCT-morphological group of patient eyes, as well as the grouping according to the presence of the tractive component. The thicker all the fovea layers in Groups 1–5 were, the worse the preoperative visual acuity was. In particular, this association was in the group of patient eyes with VMT. In Group 3, only CFT and MFT played a decisive role in preoperative visual acuity outcomes.  Table 5 provides information about the influence of the CIPLT and EIFL in Groups 3 and 1–3 on the functional parameters. There were no significant correlations among those.

In Groups 1–4, there were no correlations between the five selected quantitative fovea parameters and postoperative BCVA in contrast to the significantly negative association of the same for Group 5, but with the exception of CINLT and CIPLT. An isolated negative correlation of CONLT with postoperative BCVA for patient eyes with ERM without an OCT-apparent tractive component is noteworthy. In the same way, the increasing MFT correlated with a proportionally reduced postoperative visual acuity result in patient eyes with tractive component and in the total number of patient eyes with ERM.

In all operated patient eyes, the effect of the preoperative quantitative OCT-morphological fovea parameters on the postoperative visual acuity difference could not be demonstrated.

Table 6 presents the information of the preoperative correlations between the thickening of the fovea layers and the qualitative foveola parameters for the grouping of patient eyes according to the OCT-evident tractive component and for all groups as a whole. The RPE changes in the macular region could be divided OCT morphologically into intact (166 eyes), wavy (35 eyes), and atrophic (7 eyes), depending on the degree of severity. These showed no correlations with all investigated quantitative fovea parameters in all groupings of patient eyes, except for the negative correlation with MFT for Groups 4–5, each with tractive component. The ellipsoid zone could be OCT morphologically either intact (118 eyes) or interrupted (90 eyes). The pathological ladder correlates positively with CFT, CONLT, and CINLT for Groups 4–5. ELM-integrity is divided into intact (148 eyes), wavy (48 eyes), and interrupted (12 eyes), according to the severity of the lesions. All investigated quantitative fovea parameters except CIPLT correlated positively with ELM-integrity damage level in Groups 4–5 with tractive component. This also applies to all patient eyes for CFT and CONLT. The severity of the distortion was determined according to its depth from the inside out and divided into the next groups: none (19 eyes), only retinal nerve fiber layer (2 eyes), up to ganglion cell layer (34 eyes), up to IPL (56 eyes), and including outer plexiform layer (97 eyes). The degree of distortion of the retinal layers caused by tangential pulling forces in ERM showed a proportional positive correlation in all patient eyes, in contrast to Groups 4–5 with tractive component. Morphologically in OCT, an ERM can be either adherent (108 eyes) or separate (100 eyes). The separation of ERM correlated positively with MFT in all patient eyes as well as with CFT for Groups 1–3.

## 4. Discussion

This study presents a description of the different OCT stages of ERM with measurement of the individual retinal layers in the fovea region. As a result, a clear difference was found between the typical fovea configurations in ERM and the normal macular findings in healthy patient eyes of the same age group. The described quantitative fovea parameters could be helpful in assessing the OCT stage of ERM.

As the study by Zou et al. has shown, those with SD-OCT (Cirrus; Carl Zeiss Meditec Inc.^®^, Dublin, CA, USA) machine-measured foveal IPL, INL, and ONL thickness at ERM 42.25 ± 13.22, 75.56 ± 28.49, and 130.9 ± 22.65 *μ*m, CFT was 504.93 ± 118.14 *μ*m [22]. There was a clear difference between the fovea layers as measured mechanically in the study and manually by us in the ERM both in absolute numbers and in the ratio of the thickness of the individual fovea layers to the CFT. Theoretically, this difference could be due to the extent of the measuring range. The manual measurement of the quantitative fovea parameters was carried out only in one focal point, whereas the OCT device uses a much wider area with a diameter of about 1 mm. However, maximum visual acuity perception occurs in a very small fovea area of about 8–16 angular minutes, decreasing exponentially after distance [23]. The whole foveola is about 1.4° in size [23] so that the changes in the fovea layers considered directly in the macular center are more meaningful for the lack of visual acuity increase. Manual measurement of the thickness of the foveolar layers solves also the problem of the inaccuracy of their machine OCT morphological distribution at the epiretinal membrane.

In another study by Strasburger [24], preoperative CRT for stages 2 and 3 according to Govetto et al. and for existing MLH was 428.3 ± 63, 472 ± 71, and 371.1 ± 70 *μ*m [24]. The first two figures are similar in absolute terms to the CFT used in our study for Groups 2 and 3. The latter finding of preoperative CRT in ERM with MLH is significantly higher compared to CFT for Group 4 in our study.

In the study by Liu et al., the normal SD-OCT (Cirrus; Carl Zeiss Meditec Inc.^®^, Dublin, CA, USA) measured central subfield thickness 281.3 ± 14.5 *μ*m in 192 eyes aged 20–90 years [25]. The relevant difference to our study, in which the CFT was 221.4 ± 18.8 *μ*m, could be due both to the relatively older middle age of the patients in the control group and to the manual measurement method of CFT. According to the results of Eriksson et al., retinal thickness in the macular area decreases by 0.26–0.46 *μ*m per year with age in healthy eyes [26].

Although all quantitative fovea parameters showed a positive correlation with each other in all patient eyes with ERM, some of these (CIPLT and CINLT) could not be determined at all stages of ERM. This is because the significant ectopy of IPL and INL is observed only from the third OCT morphological stage of ERM. As a result, the measurement of IPL and INL thickness is not useable for stages 1 and 2, as well as for tractive syndromes. In contrast, CFT, MFT, and ONL thickness are universal quantitative fovea parameters for all OCT morphotypes and represent a measurement alternative.

The retrospective study by Sakai et al., with 78 vitrectomized eyes of the 76 patients with idiopathic ERM, showed a significant correlation between CFT and preoperative BCVA (*R* = 0.429, *p* < 0.001) [27]. This and the integrity of the photoreceptor layer correlated with preoperative BCVA in the prospective study by Kim et al. They examined 52 patient eyes that received a 23-gauge vitrectomy. In addition, preoperative parafoveolar INL thickness was a safe prognostic factor for postoperative BCVA [28]. Similarly, in the publication by Zou et al., foveal INL thickness correlated positively with both preoperative and postoperative BCVA [22]. Compared to our study, CFT and INL thickness were fully correlated only with preoperative BCVA in patient eye groups with tractive syndromes. However, the meta-analysis of several publications yielded controversial information about the influence of preoperative CFT on postoperative BCVA. In the systemic review by Scheerlinck et al., this was demonstrated in some studies, but in the others included in this review, there was either no correlation or only a slight association with large CFT [18]. A significant (feedback) correlation was found between preoperatively measured central INL thickness and postoperative BCVA. In contrast, there was no correlation to the central and parafoveal ONL thickness [18]. We also found no correlation between preoperative CFT and end visual acuity. In contrast, ONL thickening negatively affected postoperative BCVA in ERM without a tractive component.

In the study by Karasu and Celebi [10], 138 eyes of 106 patients with mild-to-severe EIFL were included with follow up at 3, 6, and 12 months after pars-plana vitrectomy without ILM peeling. Higher EIFL thickness was significantly correlated with lower final BCVA (*R*^2^ = 0.748, *p*=0.001). However, in contrast, there was no correlation between EIFL thickness and pre- and postoperative BCVA and postoperative visual acuity difference in our study.

According to our results, total fovea thickening, including INL and ONL, in ERM with tractive syndromes leads to lesions of the photoreceptor layers, causing the resulting metamorphopsia to develop in patients. In a publication by Ichikawa et al., INL thickness is an important biomarker in the development of ERM-accompanying metamorphopsia and causes tangential retinal disorganization [5].

## 5. Conclusions

The investigated fovea layer thicknesses (CFT, CONLT, CINLT, CIPLT, and MFT) differed significantly in all OCT morphotypes of ERM. The measurement of these layers helps to determine and describe the exact OCT-morphological stages of the ERM. The total fovea thickening in the central area proportionally influences the thickening of the individual fovea layers. The more pronounced the thickening of the fovea layers, the lower the preoperative BCVA. The preoperative BCVA in the groups of ERM with traction component showed significant correlation with CFT as well as with the thickness of individual foveal layers. For postoperative BCVA, this rule applies only in the case of the presence of an OCT-visible tractive component, in both MLH and VMT. ONL thickening can be considered as a limited prognostic factor of the absence of postoperative visual acuity improvement in ERM without a traction component. In ERM with tractive component, central foveolar retinal thickening is directly associated with loss of ellipsoid zone and ELM-integrity, which explains preoperative visual acuity reduction as well as metamorphopsia.

## Figures and Tables

**Table 1:** The distribution of cataract grading according to lens opacities classification system III (LOCS III) in OCT morphotypes of ERM with designation of the corresponding significance level (*p*-value).

OCT morphotypes	*n*	Lens Opacities Classification System III (LOCS III), mean value with standard deviation		
		Nuclear (N)	Cortical (C)	Posterior subcapsular (P)
Group 1	37	2.76 ± 0.43	1.49 ± 0.51	0.03 ± 0.16
Group 2	38	2.76 ± 0.43	1.58 ± 0.50	0.03 ± 0.16
Group 3	84	2.63 ± 0.49	1.49 ± 0.50	0.01 ± 0.11
Group 4	34	2.53 ± 0.51	1.41 ± 0.50	0.01 ± 0.10
Group 5	15	2.61 ± 0.51	1.47 ± 0.52	0.04 ± 0.17
Groups 1–5	208	2.66 ± 0.48, N ≤ 3	1.49 ± 0.50, C ≤ 2	0.02 ± 0.14, *P* ≤ 1
*p*-Value		0.169	0.724	0.574

ERM: epiretinal membrane, OCT: optical coherence tomography, and *n*: number of patient eyes.

**Table 2:** Quantitative fovea parameters for various OCT morphotypes of ERM (*p* < 0.001).

		OCT morphotypes of ERM					Control group
		Group 1	Group 2	Group 3	Group 4	Group 5	
Number of patient eyes		37	38	84	34	15	40
Quantitative fovea parameters	CFT, *µ*m	279.9 ± 61.0	395.3 ± 74.2	474.2 ± 80.7	245.4 ± 76.5	398.0 ± 168.1	221.4 ± 18.8
	CONLT, *µ*m	179.2 ± 50.5	292.1 ± 72.6	269.9 ± 61.7	167.3 ± 65.6	285.5 ± 180.1	126.9 ± 17.6
	CINLT, *µ*m	14.5 ± 3.3	16.4 ± 3.3	93.9 ± 54.8	3.1 ± 8.3	20.9 ± 11.2	18.3 ± 6.3
	CIPLT, *µ*m	0.0	0.0	28.8 ± 34.5	0.0	0.0	
	MFT, *µ*m	391.4 ± 52.0	426.8 ± 57.5	492.6 ± 70.5	439.6 ± 46.9	418.9 ± 146.4	330.2 ± 18.6

ERM: epiretinal membrane, OCT: optical coherence tomography, ONL: outer retinal nuclear layer, INL: inner retinal nuclear layer, IPL: inner plexiform retinal layer, CFT: central foveolar retinal thickness, MFT: maximum foveal retinal thickness, CONLT: central foveolar ONL thickness, CINLT: central foveolar INL thickness, and CIPLT: central foveolar IPL thickness.

**Table 3:** Pearson's correlations (*R*^2^) between the paired quantitative fovea parameters at ERM (total Groups 1–5) labeled the corresponding significance level (*p* value). ^*∗∗*^*p* < 0,01 and ^*∗∗∗*^*p* <  0,001.

Quantitative fovea parameters	CFT, *µ*m	CONLT, *µ*m	CINLT, *µ*m	CIPLT, *µ*m
CFT, *µ*m	—	0,814, *p* < 0, 001^*∗∗∗*^	0,638, *p* < 0, 001^*∗∗∗*^	0,493, *p* < 0, 001^*∗∗∗*^
CONLT, *µ*m	—	—	0,186, *p* < 0, 007^*∗∗∗*^	0,169, *p* < 0, 015^*∗∗*^
CIPLT, *µ*m	—	—	0,483, *p* < 0, 001^*∗∗∗*^	—
MFT, *µ*m	0.774, *p* < 0, 001^*∗∗∗*^	0.65, *p* < 0, 001^*∗∗∗*^	0,491, *p* < 0, 001^*∗∗∗*^	0,452, *p* < 0, 001^*∗∗∗*^

ERM: epiretinal membrane, ONL: outer retinal nuclear layer, INL: inner retinal nuclear layer, IPL: inner plexiform retinal layer, CFT: central foveolar retinal thickness, MFT: maximum foveal retinal thickness, CONLT: central foveolar ONL thickness, CINLT: central foveolar INL thickness, and CIPLT: central foveolar IPL thickness.

**Table 4:** Pearson's correlations (*R*^2^) between quantitative fovea parameters in OCT morphotypes of ERM, pre- and postoperative BCVA, and postoperative visual acuity difference with designation of the corresponding significance level (*p* value). ^*∗*^*p* < 0,05, ^*∗∗*^*p* < 0,01, and ^*∗∗∗*^*p* <  0,001.

OCT morphotypes	Visual acuity	Quantitative fovea parameters			
		CFT, *µ*m	CONLT, *µ*m	CINLT, *µ*m	MFT, *µ*m
Group 1, *n* = 36	Preoperative BCVA	−0,066, *p*=0,702	−0,038, *p*=0,824	0,084, *p*=0,625	−0,091, *p*=0,597
	Postoperative BCVA	0,168, *p*=0,329	0,149, *p*=0,386	−0,021, *p*=0,905	0,166, *p*=0,332
	Visual acuity difference	0,22, *p*=0,197	0,186, *p*=0,277	−0,064, *p*=0,713	0,231, *p*=0,176

Group 2, *n* = 37	Preoperative BCVA	−0,162, *p*=0,337	−0,146, *p*=0,388	−0,111, *p*=0,512	−0,19, *p*=0,259
	Postoperative BCVA	−0,188, *p*=0,265	−0,134, *p*=0,43	−0,092, *p*=0,587	−0,219, *p*=0,192
	Visual acuity difference	−0,056, *p*=0,743	−0,012, *p*=0,944	−0,01, *p*=0,995	−0,064, *p*=0,706

Group 3, *n* = 80	Preoperative BCVA	−0,248, *p*=0, 026^*∗*^	−0,065, *p*=0,569	−0,135, *p*=0,233	−0,318, *p*=0, 004^*∗∗*^
	Postoperative BCVA	−0,066, *p*=0,559	−0,136, *p*=0,229	0,11, *p*=0,332	−0,077, *p*=0,496
	Visual acuity difference	0,067, *p*=0,556	−0,115, *p*=0,309	0,186, *p*=0,099	0,102, *p*=0,369

Group 4, *n* = 33	Preoperative BCVA	−0,116, *p*=0,519	−0,08, *p*=0,658	−0,005, *p*=0,978	−0,355, *p*=0,053
	Postoperative BCVA	0,045, *p*=0,803	0,025, *p*=0,891	−0,162, *p*=0,369	0,076, *p*=0,673
	Visual acuity difference	0,02, *p*=0,91	0,013, *p*=0,944	−0,192, *p*=0,284	0,167, *p*=0,353

Group 5, *n* = 15	Preoperative BCVA	−0,852, *p* < 0, 001^*∗∗∗*^	−0,763, *p* < 0, 001^*∗∗∗*^	−0,683, *p* < 0, 001^*∗∗∗*^	−0,799, *p* < 0, 001^*∗∗∗*^
	Postoperative BCVA	−0,604, *p*=0, 017^*∗*^	−0,534, *p*=0, 04^*∗*^	−0,466, *p*=0,08	−0,687, *p*=0, 005^*∗∗*^
	Visual acuity difference	−0,07, *p*=0,804	−0,052, *p*=0,855	−0,03, *p*=0,915	−0,236, *p*=0,397

Groups 1–3, *n* = 153	Preoperative BCVA	−0,218, *p*=0, 007^*∗∗*^	−0,163, *p*=0, 047^*∗*^	−0,112, *p*=0,169	−0,254, *p*=0, 002^*∗∗*^
	Postoperative BCVA	−0,068, *p*=0,402	−0,163, *p*=0, 044^*∗*^	0,086, *p*=0,289	−0,051, *p*=0,531
	Visual acuity difference	0,052, *p*=0,522	−0,068, *p*=0,407	0,15, *p*=0,064	0,097, *p*=0,234

Groups 4–5, *n* = 48	Preoperative BCVA	−0,503, *p* < 0, 001^*∗∗∗*^	−0,499, *p* < 0, 001^*∗∗∗*^	−0,329, *p*=0, 024^*∗*^	−0,528, *p* < 0, 001^*∗∗∗*^
	Postoperative BCVA	−0,259, *p*=0,078	−0,27, *p*=0,066	−0,189, *p*=0,204	−0,413, *p*=0, 004^*∗*^
	Visual acuity difference	0,087, *p*=0,563	0,069, *p*=0,643	0,032, *p*=0,828	−0,077, *p*=0,606

ERM: epiretinal membrane, OCT: optical coherence tomography, BCVA: best-corrected decimal far visual, ONL: outer retinal nuclear layer, INL: inner retinal nuclear layer, IPL: inner plexiform retinal layer, CFT: central foveolar retinal thickness, MFT: maximum foveolar retinal thickness, CONLT: central foveolar ONL thickness, CINLT: central foveolar INL thickness, CIPLT: central foveolar IPL thickness, and *n*: number of patient eyes.

**Table 5:** Pearson's correlations (*R*^2^) between CIPLT and EIFL in OCT morphotypes of ERM, pre- and postoperative BCVA, and postoperative visual acuity difference with designation of the corresponding significance level (*p*-value). *∗p* < 0,05.

OCT morphotypes	Visual acuity	CIPLT, *µ*m	EIFL, *µ*m
Group 3, *n* = 80	Preoperative BCVA	−0,214, *p*=0,057	−0,216, *p*=0,124
	Postoperative BCVA	−0,084, *p*=0,458	0,046, *p*=0,688
	Visual acuity difference	0,048, *p*=0,671	0,174, *p*=0,124

Groups 1–3, *n* = 153	Preoperative BCVA	−0,169, *p*=0, 038^*∗*^	−0,148, *p*=0,067
	Postoperative BCVA	−0,029, *p*=0, 718^*∗*^	0,054, *p*=0,506
	Visual acuity difference	0,068, *p*=0,404	0,142, *p*=0,081

ERM: epiretinal membrane, OCT: optical coherence tomography, BCVA: best-corrected decimal far visual, IPL: inner plexiform retinal layer, CIPLT: central foveolar IPL thickness, EIFL: ectopic inner foveal layers, and *n*: number of patient eyes.

**Table 6:** Pearson's correlations (*R*^2^) between quantitative fovea parameters and qualitative macular changes in OCT morphotypes of the ERM with designation of the corresponding significance level (*p*-value). ^*∗*^*p* < 0,05, ^*∗∗*^*p* < 0,01, and ^*∗∗∗*^*p* < 0,001.

OCT morphotypes	Qualitative macular changes	Quantitative fovea parameters				
		CFT, *µ*m	CONLT, *µ*m	CINLT, *µ*m	CIPLT, *µ*m	MFT, *µ*m
Groups 1–3, *n* = 159	RPE lesions	0,005, *p*=0,946	−0,098, *p*=0,221	−0,046, *p*=0,564	−0,08, *p*=0,313	−0,006, *p*=0,942
	Integrity of ellipsoid zone	0,131, *p*=0,1	0,068, *p*=0,396	−0,001, *p*=0,988	−0,046, *p*=0,568	0,146, *p*=0,066
	ELM integrity	0,061, *p*=0,442	−0,071, *p*=0,372	0,069, *p*=0,388	−0,065, *p*=0,419	0,057, *p*=0,476
	Distortion of the retinal layers	0,292, *p* < 0, 001^*∗∗∗*^	0,248, *p*=0, 002^*∗∗*^	0,164, *p*=0, 039^*∗*^	0,113, *p*=0,154	0,287, *p* < 0, 001^*∗∗∗*^
	Separation of ERM	0,168, *p*=0, 034^*∗*^	0,094, *p*=0,24	0,106, *p*=0,184	0,066, *p*=0,406	0,187, *p*=0, 018^*∗*^

Groups 4–5, *n* = 49	RPE lesions	−0,163, *p*=0,262	−0,238, *p*=0,1	−0,015, *p*=0,919	—	−0,284, *p*=0, 048^*∗*^
	Integrity of ellipsoid zone	0,342, *p*=0, 016^*∗*^	0,293, *p*=0, 041^*∗*^	0,315, *p*=0, 027^*∗*^	—	0,165, *p*=0,258
	ELM integrity	0,548, *p* < 0, 001^*∗∗∗*^	0,514, *p* < 0, 001^*∗∗∗*^	0,579, *p* < 0, 001^*∗∗∗*^	—	0,285, *p*=0, 047^*∗*^
	Distortion of the retinal layers	−0,024, *p*=0,868	−0,007, *p*=0,962	−0,151, *p*=0,3	—	0,384, *p*=0, 006^*∗∗*^
	Separation of ERM	0,151, *p*=0,3	0,169, *p*=0,244	−0,035, *p*=0,811	—	0,397, *p*=0, 005^*∗∗*^

ERM: epiretinal membrane, OCT: optical coherence tomography, RPE: retinal pigment epithelium, ELM: external retinal limiting membrane, ONL: outer retinal nuclear layer, IN: inner retinal nuclear layer, IPL: inner plexiform retinal layer, CFT: central foveolar retinal thickness, MFT: maximum foveal retinal thickness, CONLT: central foveolar ONL thickness, CINLT: central foveolar INL thickness, CIPLT: central foveolar IPL thickness, and *n*: number of patient eyes.

## Data Availability

The data used to support the findings of the study can be obtained from the corresponding author upon request.

## Abbreviations

ERM: Epiretinal membrane
OCT: Optical coherence tomography
BCVA: Best-corrected decimal far visual acuity
RPE: Retinal pigment epithelium
OS/IS junction: Connection between the outer and inner segments of the photoreceptors
ELM: External retinal limiting membrane
ILM: Inner retinal limiting membrane
ONL: Outer retinal nuclear layer
INL: Inner retinal nuclear layer
IPL: Inner plexiform retinal layer
CFT: Central foveolar retinal thickness
MFT: Maximum foveal retinal thickness
CONLT: Central foveolar ONL thickness
CINLT: Central foveolar INL thickness
CIPLT: Central foveolar IPL thickness
DRIL: Disorganization of the inner retinal layers
EIFL: Ectopic inner foveal layers.
